# Cloud-Based Influenza Surveillance System in Emergency Departments Using Molecular-Based Testing: Advances and Challenges

**DOI:** 10.5811/westjem.2021.9.52741

**Published:** 2022-02-14

**Authors:** Kathryn Shaw-Saliba, Bhakti Hansoti, Howard Burkom, Diego A. Martinez, Anna DuVal, Brian Lee, Phong Chau, Breanna McBride, Yu-Hsiang Hsieh, Vidiya Sathananthan, David Persing, Michael Turnlund, Roxanne Shively, Andrea Dugas, Richard E. Rothman

**Affiliations:** * Department of Emergency Medicine, Johns Hopkins University School of Medicine, Baltimore, Maryland; † Applied Physics Laboratory, Johns Hopkins University, Laurel, Maryland; ‡ School of Industrial Engineering, Pontificia Universidad Católica de Valparaíso, Valparaíso, Chile; § Office of the Chief Information Officer, Office of the Chief Operating Officer, Centers for Disease Control and Prevention, Atlanta, Georgia; ¶ Department of Engineering and Software, Cepheid, Sunnyvale, California; || Biomedical Advanced Research and Development Authority, US Department of Health and Human Services, Washington, District of Columbia; # Johns Hopkins University School of Medicine, Department of Emergency Medicine, Baltimore, Maryland; ** National Emergency Department Influenza Consortium Site Leads: Greg Moran, David Talan (UCLA Olive View, Sylmar California), Frank Lovecchio (Maricopa Medical Center, Phoenix, Arizona), Mark Steele (Truman Medical Center, Kansas City, Missouri)

## Abstract

**Introduction:**

Electronic influenza surveillance systems aid in health surveillance and clinical decision-making within the emergency department (ED). While major advances have been made in integrating clinical decision-making tools within the electronic health record (EHR), tools for sharing surveillance data are often piecemeal, with the need for data downloads and manual uploads to shared servers, delaying time from data acquisition to end-user. Real-time surveillance can help both clinicians and public health professionals recognize circulating influenza earlier in the season and provide ongoing situational awareness.

**Methods:**

We created a prototype, cloud-based, real-time reporting system in two large, academically affiliated EDs that streamed continuous data on a web-based dashboard within hours of specimen collection during the influenza season. Data included influenza test results (positive or negative) coupled with test date, test instrument geolocation, and basic patient demographics. The system provided immediate reporting to frontline clinicians and to local, state, and federal health department partners.

**Results:**

We describe the process, infrastructure requirements, and challenges of developing and implementing the prototype system. Key process-related requirements for system development included merging data from the molecular test (GeneXpert) with the hospitals’ EHRs, securing data, authorizing/authenticating users, and providing permissions for data access refining visualizations for end-users.

**Conclusion:**

In this case study, we effectively integrated multiple data systems at four distinct hospital EDs, relaying data in near real time to hospital-based staff and local and national public health entities, to provide laboratory-confirmed influenza test results during the 2014–2015 influenza season. Future innovations need to focus on integrating the dashboard within the EHR and clinical decision tools.

## INTRODUCTION

There are approximately 140,000–710,000 hospitalizations and 12,000–56,000 deaths from seasonal influenza, and influenza-related cases occur annually in the US.^1^,^2^ The severity of a given influenza season varies widely and is determined by a myriad of factors, including characteristics of circulating viruses, the timing of the season, vaccine utilization, and vaccine effectiveness.^2^,^3^ Physician awareness of the diagnosis associated with rapid influenza diagnostics has been shown to significantly reduce the number of laboratory tests and radiographs ordered. Furthermore, diagnostic awareness is associated with decreased antibiotic use rates, increased use of antivirals, and decreased length of time to discharge from the emergency department (ED).^4^,^5^

Given the seasonality of influenza, real-time situational awareness serves to inform clinical decision-making and helps with preparedness within the institution and community, as well as nationally. For example, in response to the US Centers for Disease Control and Prevention (CDC) efforts to strengthen global influenza surveillance, 39 countries participated in a program that strengthened their ability to use national influenza data in decision-making in several ways: 1) drive updates to national pandemic preparedness plans; 2) create evidence-based vaccine guideline; 3) determine the best use of antiviral medications; and 4) determine the need for community mitigation measures such as school closures.^6^,^7^

The CDC tracks the severity of each influenza season through compilation and analysis of various data types, including laboratory test results from respiratory specimens (virologic surveillance); outpatient reports of influenza-like illness (ILI) (syndromic surveillance); influenza-associated deaths (mortality surveillance); laboratory-confirmed influenza-associated hospitalizations (hospitalization surveillance); and estimated geographic spread of influenza activity by state (geographic surveillance).^8^,^9^ This data is collected from disparate organizations and then sent to and collated by the CDC. Notably, a systematic review of ED-based surveillance reported delays of up to 21 days before sentinel surveillance data is shared and up to 24 days before virological confirmation, which reports data with age stratification.^10^ Even cloud-based surveillance strategies, such as Wikipedia, have intrinsic delays of up to 10 days, related in part to the need for manual data uploads.^11^

Reducing reporting lag-time is key to real-time surveillance. Two reporting systems based on ILI have been described: the autoregressive electronic health record support vector machine (ARES), which couples ILI visit data with historical patterns of influenza activity; and Flu Near You, a crowdsourcing method that relies on self-reported ILI.^12^,^13^ Even though both systems report real-time data, their reliance on ILI (vs direct laboratory diagnostic test data) is limiting, given that the influenza cases reported have not been confirmed. Recently, several cloud-based reporting systems, which take advantage of newer, real-time molecular diagnostic assays, have been reported.^6^,^14^ These systems represent an important step forward since they permit more timely aggregate molecular diagnostic assay results. Limitations still remain, including lack of field testing of these systems in EDs and limitations in data reporting (ie, lack of incorporation of demographic data in association with influenza test results).^6^,^7^,^15^,^16^

**Population Health Research Capsule**

What do we already know about this issue?

*Real-time pandemic surveillance is necessary and important to support clinical decision-making.*

What was the research question?

*What are the structural challenges to connecting laboratory results to clinical data and making it available to end-users in real time?*

What was the major finding of the study?

*An essential services for interoperability framework can be used effectively to overcome design challenges, to build solutions that are effective across facilities.*

How does this improve population health?

*Real-time acess to surveillance data can aid clinical and public health decision-making to improve patient outcomes and inform preparedness activities.*

Given the above shortfalls, investigators at Johns Hopkins University worked with scientists at Cepheid (Sunnyvale, CA), a molecular diagnostics company, and the Biomedical Advanced Research and Development Authority of Health and Human Services (BARDA/HHS) to create a prototype cloud-based, real-time influenza surveillance system, which was implemented in four US EDs. The system was designed with the expressed goal of integrating data from ED triage with near point-of-care molecular testing from multiple EDs, using a data aggregation platform that permitted sharing basic demographic data elements from the electronic health record (EHR). Data was made immediately available to relevant end-users (ie, frontline clinicians, hospital administrators, and local and national public health entities) on a secure, web-based interface. In this report, we describe the methods and challenges encountered with the creation of a multicenter, cloud-based influenza reporting system and discuss opportunities for future development.

## METHODOLOGY

### Case Description

This is a case study of a prototype integrated surveillance strategy that combines molecular testing data with clinical demographics. The objective of this pilot system was to build a data management and connectivity process across multiple data systems while also linking multiregional EDs, their neighboring health departments, and epidemiologists at the CDC. The secondary objective of this activity was to document the process for system integration and develop a blueprint for implementation. The overarching goal is to enable aggregation and interpretation of data across multiple sites for real-time molecular surveillance of influenza employing a cloud-based reporting system. This pilot system leveraged the National Emergency Department Influenza Consortium made up of four large, urban academically affiliated adult EDs: Johns Hopkins Hospital (JHH) (Baltimore, MD); Truman Medical Center (TMC) (Kansas City, Mo); Maricopa Medical Center (MMC) (Phoenix, AZ); and Olive View-UCLA Medical Center (UCLA) (Sylmar, CA).

Here we first describe the source data and then the regulatory and practical steps employed in pre-implementation and implementation, and review implementation challenges. The study was approved by the institutional review boards (IRB) at each site as an exempt study. The procedures within this activity were deemed a part of a quality improvement initiative.

### Source Data

The study was conducted over two consecutive influenza seasons. The system was first developed and refined with feedback from end-users at two lead sites, JHH and UCLA, during the 2013–2014 influenza season. The final version of the system was subsequently rolled out at all four sites (adding TMC and MMC) during the 2014–2015 influenza season and included a clinical decision guideline that was integrated into each hospital’s EHR to guide influenza testing, with sharing of demographic data into the cloud.^17^ A comparison of the sites is provided in Table 1. All adult patients presenting to these EDs were systematically screened by nurses during triage. Nasopharyngeal specimens were collected from patients who met screening criteria and were immediately sent to the onsite laboratory for testing with Cepheid’s on-demand molecular Xpert Flu assay, a rapid and highly sensitive polymerase chain reaction-based assay approved by the US Food and Drug Administration, which permitted differentiation of influenza A, B, and 2009 H1N1.^18^,^19^ The test instruments were connected to a cloud-based data aggregation system with a secure, web-based interface allowing for the automatic upload of date and time of test completion and geolocation of the test instrument in addition to test results.

### Pre-implementation: Site Preparation and Approvals

**Figure 1:**
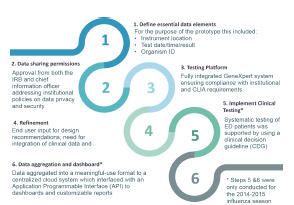
Preparatory steps for surveillance implementation.

The establishment of this prototype cloud-based influenza surveillance system required a series of preparatory steps (Figure 1) carried out across both influenza seasons. A participatory design approach was used to determine a priori end-user input for design recommendations to maximize the system utility during the 2013–2014 season at the two lead sites. Using an iterative approach, the system was then refined to include the collation of demographic data for the 2014–2015 flu season across all four sites. Each site obtained legal approval from their institutional information security office and their IRBs to permit sharing of de-identified project data with designated clinical and external public health partners’ sites. Each site also worked with a representative from Cepheid to install the GeneXpert platform, creating the pathway for communicating GeneXpert test results with the laboratory information system software (LIS) and verifying local, secure, web-based connections between the GeneXpert platform and the RemoteXpert cloud.

### Implementation: Data Aggregation and Visualization

**Figure 2:**
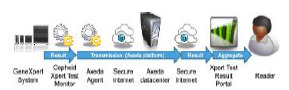
Data flow for cloud-based data aggregation system.

**Figure 3:**
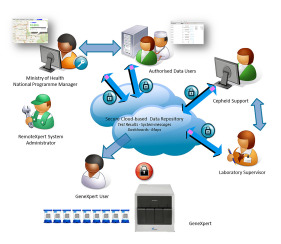
Schematic of architecture and data flow for the operational pilot surveillance system (RemoteXpert).

Data from the Cepheid GeneXpert platform was automatically uploaded to the RemoteXpert cloud within two hours of specimen collection, and made immediately available via a web-based interface to affiliated end-users who were part of the study (Figure 2). The aggregated data in the RemoteXpert was stored in a secure, third-party hosting facility, which met the required national data security and protection guidelines and included failover features to minimize system downtime. An operational overview of the system is provided in Figure 3.

**Figure 4:**
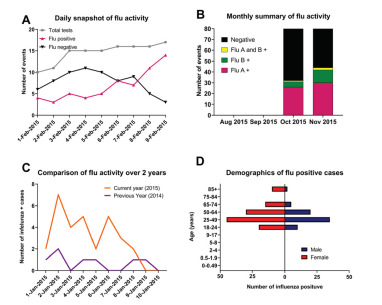
Rendering of the key data elements available on the RemoteXpert dashboard.

The RemoteXpert interface displayed full test results, including an interactive map with testing results by geographic location, test instrument, and other visualizations of results by subtype and location. Test results from the four EDs allowed real-time analysis of regional differences in disease burden and identified where current epidemics were occurring. Figure 4 shows a representation of each component of the dashboard, including the geocoding of the sites and a trendline of testing volume and result (Figure 4 Panel 1), a snapshot of testing results aggregated by demographics (Figure 4 Panel 2), a snapshot of influenza activity (Figure 4 Panel 3), and a trendline that compared current vs historical influenza activity (Figure 4 Panel 4). For more in-depth analyses, a “medical dashboard” was created, which permitted end-users to customize views of aggregated data. The portal provided specific instructions for formatting, displaying, and downloading data for local analysis. Additionally, full test-result listings with associated data were available for download in .csv format at any time, depending on end-user privileges.

The insert below (Figure 4) shows an example rendering of the data visualizations that includes the following: A. Daily summary of influenza activity; B. Monthly summary of influenza; C. Comparison of influenza daily activity over two years: and D. Demographics of positive influenza cases. In the pilot dashboard (data was provided by clinical sites) we were able to provide daily test results from the previous 14 days, as well as comparative data from the previous two years and the prior month; this allowed clinicians to quickly identify trends and frame with historical perspective. It was also possible to aggregate data by flu type and demographics, which we believe would influence clinical decision-making.

Access privileges to the system’s web-based interface were defined at each site as follows: 1) “*Superusers*,” the principal investigators and program manager; 2) “*Supernational readers*,” staff at JHU, the BARDA, and CDC, who could access data from all sites; 3) *“Site coordinators*” with access to local data, test results, and local user sign-on information; and 4) “*Hospital-based users.*” Access control software provided each user one or more profiles, each associated with specific permissions. Profiles were established, and associated privileges were enabled when each user logged in.

## RESULTS

The pilot cloud-based influenza surveillance system successfully displayed in near real-time a combined total of 5937 test results, including 1070 type-specific influenza positives, with associated geolocation, date, and time. Cumulative data on the implementation of the pilot cloud-based influenza surveillance system during the 2014–2015 season at all four study sites is provided in Table 2.

Strategies were implemented to manage the interoperability issues based on the Office of the National Coordinator for Health Information Technology (ONC) of the US Department of Health and Human Services, a summary of which is provided in Table 3. The ONC specifically seeks to enable an interoperable health information technology ecosystem that makes the “right data available to the right people at the right time” to support advances in access to care, quality of care, clinical awareness, and public health situational awareness.

## DISCUSSION

We created and implemented a prototype influenza surveillance system that relied on molecular testing from triage workflow across multiple EDs and shared data in real time on a cloud-based server. When this project began, coupling laboratory-based influenza molecular tests with cloud-based reporting systems was in the earliest development phases. Since that time, two systems that specifically integrate respiratory molecular virology testing with cloud-based dashboards for clinicians and local public health users have been described.^6^,^14^ Both systems, as well as the one presented here, represent an advancement in influenza surveillance methodology, decreasing the two-week lag time associated with traditional influenza surveillance approaches. The system’s intrinsic architectural design guarantees immediate delivery of molecular testing data (herein, from the GeneXpert device) to the varied end-users and eliminates any lag-time delays. The test result, date/time, and geolocation are continually pushed from the GeneXpert database to the RemoteXpert cloud, making test data available to the web application in real time.

One of the major obstacles we encountered was associated with institutional permissions (from the chief information officer) for sending basic demographic data (including gender, age group, and race) into the cloud. Furthermore, hospitals encountered unanticipated technical software connectivity challenges, preventing fully automated data feeds. In addition, while the software engineers from Cepheid were able to interface the GeneXpert instrument with the LIS, a technical obstacle was discovered when attempting to transmit LIS information directly into the cloud. An intermediate software solution was used as a bridge to address that issue, permitting sites to manually pair demographic data elements with GeneXpert within the Xpert Reporter client user interface in real time prior to sending data into the cloud. Notably, since the time of this study, new software connectivity solutions have been designed (including that developed by Cepheid, coined “Cepheid 360 Sync”), which permits fully automated data feeds with a synchronized transfer of both demographic and molecular test directly into the cloud. This C360 system is now made available for all Cepheid GeneXpert customers as an embedded feature of the GeneXpert software, which comes with the set-up of the Cepheid instrument. The software includes information for laboratory customers to configure how much Protected Health Information (PHI) will be uploaded to the C360 cloud, which can be used both for disease monitoring and advanced analysis based on the specific needs of the end-user. Testing and evaluation of these approaches for research and public health use are now underway.

One notable limitation of the system we describe here was the requirement that end-users log on and access the dashboard. For this prototype evaluation, we did not systematically monitor how frequently that occurred. Nevertheless, informal feedback from users, including the public health stakeholders, was consistently positive, with many indicating that the system filled an important gap associated with timely information-sharing between disparate healthcare organizations. Future innovation should consider removing the requirement for a separate log-in with institutional permissions and/or delivering push notifications to the clinical workspace, ie, provide specific alerts to end-users when pre-specified influenza risk thresholds have been reached. Another limitation of this prototype system that challenged sustainability and uptake is that data access and sharing between local public health users and the CDC required extensive discussion with leadership at each site and the establishment of short-term agreements under a study protocol between entities at the time this project was carried out. Accordingly, while end-users (both clinician and public health) expressed interest in retaining the capability of the cloud-based reporting system, none did so.

Since the time this study was carried out, there have been notable advances in that there are now better established and streamlined pathways for end-user permissions under local, state, and federal jurisdictions.^21^ Informally, end-user feedback indicated that one of the most valuable aspects of the system was the ease of use of the simple dashboard display. Most recently, we have seen with the advent of COVID-19 the rapid popularity of the Johns Hopkins University’s COVID-19 dashboard, a user-friendly tool designed to track the outbreak as it unfolds. Here, all data collected and displayed have been made freely available initially through Google Sheets and later through a GitHub repository, along with the feature layers of the dashboard, which are now included in the Esri Living Atlas.^22^,^23^

Looking forward, the Centers for Medicare & Medicaid Services now requires hospitals to electronically share data, including laboratory results and syndromic surveillance data, with local, state, and federal agencies.^24^,^25^ As observed in this study, however, hospitals can still experience technical and administrative barriers to rapid, timely sharing of information, and when seasonal illness rapidly surges, these delays may prove to be fatal flaws. With COVID-19, some advances have been implemented with multiple public health surveillance actions developed to improve detection of severe acute respiratory syndrome coronavirus (SARS-CoV-2) in the US, permitting tracking of its spread, in part through the establishment of national surveillance case definition with the addition of coronavirus disease to the list of notifiable conditions. Challenges in conducting effective case-based surveillance and the public health data supply chain remain, however, and have in part resulted in the relatively slow uptake of public health measures and ultimately failure to effectively curb the pandemic.^26^ The ongoing information gaps stand as challenges to the clinical and public health response to influenza and other emerging pandemics. Now is the time and opportunity to implement meaningful surveillance from the frontlines of the healthcare system.

## LIMITATIONS

In this paper we successfully present a case study of the implementation of a modular, access-based, secure, real-time influenza surveillance system. We did not seek to evaluate the implementation of the pilot integrated surveillance system and thus are unable to make assertions about the end-user experience, integration, and sustainability measures or impact on patient outcome. Furthermore, since the piloting of this system, several advances to the platforms themselves have been made, as noted in the discussion. Nevertheless, given the recent experience of the COVID-19 pandemic, a renewed focus on real-time surveillance strategies and an understanding of the system challenges to achieve this goal is needed.

## CONCLUSION

The principal outcome of this project was the successful implementation of a prototype distributed, modular, access-based, secure, real-time influenza surveillance system that coupled molecular diagnostic test data with basic demographics. This system effectively integrated multiple data systems at four distinct hospital EDs, relaying data in near real time to hospital-based staff and local and national public health entities, which afforded timely local situational awareness of laboratory-confirmed influenza test results during the 2014–2015 influenza season. Future implementation strategies should focus on harnessing the full power of real-time regional and national influenza situational awareness, which could include push notifications to clinical decision-makers using adaptive clinical decision guidelines informed by surveillance data, and to public health professionals to provide more timely monitoring and response to influenza. This cloud-based surveillance system yields data that is particularly useful for identifying the start of the influenza season, guiding surge management, and informing outbreak response in the event of a pandemic season, and could easily be adapted for other respiratory virus surveillance, such as SARS-COV-2.

## Supplementary Information



## Figures and Tables

**Table 1:** Comparison of demographics, EMR, testing strategy and reporting by study sites.

Facility	Annual volume	Electronic health record	Influenza testing strategy pre-implementation	Data reporting pre-implementation
Site 1	66,000	EPIC	Physician gestalt, ie, testing based on the clinical determination of the treating physician (resident or attending).	Weekly reporting to Maryland DOH
Site 2	65,000	ORCHID		Weekly reporting to LAC DPH
Site 3	60,000	EPIC		Weekly report AZ DHS
Site 4	62,000	Cerner		Weekly reporting to KDHEKS

*DOH*, Department of Health; *LAC DPH*, Los Angeles County Department of Public Health; *AZ DHS*, Arizona Department of Health Services; *KDHEKS*, Kansas Department of Health & Environment.

**Table 2:** Cumulative data from the pilot implementation of the integrated influenza surveillance system.

	All sites	JHU	UCLA	MMC	TMC
Patients through department	126,539	33,500	42,091	24,681	26,267
Patients assessed by CDG N(%)	118,916 (94%)	30,516 (91%)	38,741 (92%)	23,603 (96%)	26,056 (99%)
Patient who met criteria N(%)	6,955 (6%)	2,079 (7%)	2,582 (7%)	1,368 (6%)	926 (4%)
Xpert Flu tests ordered N(%)	6,601 (95%)	2,000 (96%)	2,362 (91%)	1,313 (96%)	926 (100%)
Specimens collected N(%)	5,939 (90%)	1,710 (86%)	2,019 (85%)	1,284 (98%)	926 (100%)
Tests resulted N(%)	5,937 (100%)	1,710 (100%)	2,017 (100%)	1,284 (100%)	926 (100%)
Test results appearing in EHR N(%)	5,937 (100%)	1,710 (100%)	2,017 (100%)	1,284 (100%)	926 (100%)
Patients positive for influenza N(%)	1,070 (18%)	323 (19%)	367 (18%)	202 (16%)	178 (19%)

*JHU*, Johns Hopkins University; *UCLA*, University of California, Los Angeles; *MMC*, Madras Medical College, *TMC*, Texas Medical center; *CDG*, clinical decision guideline; *EHR*, electronic health record.

**Table 3:** Management of interoperability issues based on guiding principles of the Office of the National Coordinator for Health Information Technology.

ONC’s essential services for interoperability	Project-specific task requirement	Issue	Management of issue
Accurately match individuals, providers, and their information across data sources.	Bi-directional data transfer to combine influenza test result, time, the location from GeneXpert with demographics	Required demographic details were stored in different systems (eg, LIS, EHR)	Data were manually entered by research coordinators into a laptop containing the test result data, combined, and uploaded to a cloud-based interface by means of Cepheid software.
Directories of the technical and human-readable endpoints for data sources, so they and the respective data are discoverable.	Provide access to test results from cloud-based systems to distributed user locations	No natural interface because of differences among users’ proprietary systems.	Users accessed the system through a password-protected Cepheid site, where they could also designate upload destinations for further analysis.
Authorizing users to access data from the data sources	Provide end-users with a hierarchy of access privileges	None	A logical hierarchy of access privileges.
Authenticating users when they want to access data from data sources	Control access with individual user accounts	A mass invitation provided by Cepheid was rejected by some site firewalls	Access control and definitions were handled via the CanCan Ruby library, which provided a Declarative Authentication DSL for specifying model permissions and enforcing them at the controller level.
Securing the data when it is stored or maintained in the data sources and in transit, ie, when it moves between source and user	De-identified data must remain secure during transmission between local testing site and cloud-based system.	None	Test results entering the Cepheid cloud portal via a secured transport layer security channel were processed by a test results processor service to generate non-sensitive aggregations that the cloud software could leverage for analysis, visualization, technical support, and other administrative functions.
Representing data at a granular level to enable reuse	Transmit to CDC database using HL7 code and be accessible to other end-users via dashboards and comma-delimited files	Visualization and detailed data needs varied among users.	Dashboard designs for visualization were adapted from previous Cepheid systems and made available through a secure website in the Cepheid cloud. Designs were adapted to the needs of influenza surveillance users; for example, a “medical dashboard” allows inspection of data aggregated by location and laboratory.
Handling information from varied information sources in both structured and unstructured formats	The cloud-based system had to receive both test results and demographic data in available formats.	Demographic data were represented in different formats across user sites.	The Cepheid software for uploading and managing data accepted only structured data records; the correct structure from GeneXpert test results was automatic. However, site coordinators had to ensure formatting and completeness of demographic details merged from the LIS systems.

*ONC*, Office of the National Coordinator for Health Information Technology; *LIS*, laboratory information system software; *EHR*, electronic health record; *DSL*, domain-specific language.
