# Supplementary material for: Cloud-Based Influenza Surveillance System in Emergency Departments Using Molecular-Based Testing: Advances and Challenges
Source: West J Emerg Med. 2022 Feb 14;23(2):115–23. doi: 10.5811/westjem.2021.9.52741 (PMC8967469; doi:10.5811/westjem.2021.9.52741)
Supplement: Supplementary file 1 [file wjem-23-115-s001.docx]

**Supplementary**

**Figure S1. Schematic of the RemoteXpert cloud architecture and the functional data flow.** The RemoteXpert cloud is split into two components: 1) client (Xpert Reporter) and (2) server. Once the GeneXpert test is run, results are uploaded to the Xpert Reporter client, which has two functional components: the Reporter client user interface (UI) and the reporter daemon service. The UI provides a functional interface for users to configure the daemon, manage additional data (ie, demographics), and view the status of test uploads. However, the UI does not communicate with any databases or servers but only serves as a two-way link channel with the daemon. The daemon runs on the local GeneXpert system and handles Xpert Reporter operations, including communication with the receiver server, connection to the GeneXpert database, and persistence of local data in an encrypted SQLite database. The daemon runs and continuously communicates with the server. The server (number 2 in Fig. 1) has multiple components—the communication layer and the processing layer. In addition to managing communication with the client, the receiver server receives and processes tests and provides an endpoint for live data updates. The receiver server then communicates with the relational database containing all the data stored relationally for the web application and with the user interface containing the dashboard. The receiver server also communicates with the ElasticSearch Indexing Engine, which stores test results and is used for fast listings and aggregation of tests in both index and dashboard pages for the web application. The web application performs queries on available data and is a set of independent processes running on Apache that can handle long-running jobs, such as .csv generation, and operates with the Poirot web inspector for data visualizations. The server can be scaled at various points, including the ElasticSearch and web application, to increase availability and performance.

**Supplementary S2: Influenza testing and treatment clinical decision guideline (CDG)**
